# The Effects of Maekmoondong-Tang on Cockroach Extract-Induced Allergic Asthma

**DOI:** 10.1155/2014/958965

**Published:** 2014-03-03

**Authors:** Soojin Park, Sung-Hwa Sohn, Kyung-Hwa Jung, Kun-young Lee, Yu Rim Yeom, Gae-Eun Kim, Sungki Jung, Heejae Jung, Hyunsu Bae

**Affiliations:** ^1^Department of Physiology, College of Korean Medicine, Kyung Hee University, Number 1 Hoegi-dong, Dongdaemungu, Seoul 130-701, Republic of Korea; ^2^Division of Allergy and Respiratory System, College of Korean Medicine, Kyung Hee University, Seoul 130-701, Republic of Korea

## Abstract

Maekmoondong-tang (MMDT) has long been used in Asian countries to treat respiratory diseases. However, the precise mechanisms underlying its effects on asthma are unknown. This study was conducted to evaluate the protective effects of MMDT in a cockroach allergen (CKA-)induced animal model of allergic asthma. After being challenged with CKA, the number of macrophages, eosinophils, neutrophils, lymphocytes, and total cells in the bronchoalveolar lavage fluid (BALF) was evaluated. The Th2 specific cytokines IL-4, IL-5, and IL-13 were also analyzed in BALF along with IgE levels in serum. For histological analysis, hematoxylin and eosin (H&E) staining, periodic acid-Schiff (PAS) staining, and immunohistochemical staining were performed. In addition, airway hyperresponsiveness was assessed by noninvasive plethysmography. The cellular profiles and histopathologic analysis demonstrated that peribronchial and perivascular inflammatory cell infiltrates were significantly decreased in the MMDT-treated groups compared with the cockroach extract-injected (CKA) groups. In addition, the IgE, IL-4, IL-5, and IL-13 levels were significantly decreased in the MMDT group. MMDT treatment also significantly attenuated airway hyperresponsiveness. These results demonstrated that MMDT significantly reduced the hallmark signs of asthma: elevated serum IgE, airway eosinophilia, airway remodeling, mucus hypersecretion, and airway hyperresponsiveness. The remarkable antiasthmatic effects of MMDT suggest its therapeutic potential in allergic asthma treatment.

## 1. Introduction

Approximately 300 million people currently suffer from asthma, and approximately 180,000 deaths are associated with asthma each year [1, 2]. Asthma is one of the most common chronic disorders of the airways and affects adults and children of all ages [1, 3, 4]. Recently, asthma has been increasing disproportionately in densely populated urban areas, where large numbers of cockroaches can be found. Specifically, cockroach allergen (CKA) exposure has demonstrated a significant correlation with a rise in childhood and adolescent asthma [5, 6]. Allergic asthma death is preventable. However, current standard medication therapies can produce potential negative side effects, such as retardation of growth, induction of insulin resistance, loss of bone mass, and suppression of the immune system, and do not consistently ameliorate airway inflammation in many asthmatic individuals [3, 7, 8]. Therefore, there is a need for the development of safe and efficacious treatments [9, 10].

Maekmoondong-tang (MMDT) has been used to treat respiratory diseases such as emphysema, bronchitis, and asthma in Asia for thousands of years. There have been many *in vitro* [11] and *in vivo* [12] studies to identify the potential efficacy of MMDT in asthma. In experimental studies, MMDT reduced malformed respiration and eosinophil infiltration and released the tension of bronchial smooth muscle [13, 14]. Additionally, MMDT had immunomodulatory action and regulated many immune cytokines to reduce hypersensitivity [11, 12, 15]. In clinical studies, patients who were treated with MMDT had increased FEV_1_, improved symptom scores [16], and decreased cough sensitivity [17].

Although there have been numerous studies on MMDT, the therapeutic effects and underlying mechanisms of how MMDT functions to ameliorate pulmonary disorders have not been evaluated. To elucidate the mechanism of how MMDT modulates the CKA-induced allergic response, we evaluated the alteration of immune cells, inflammatory cytokine secretion, and IgE level in bronchoalveolar lavage fluid (BALF) and performed histological examinations in a CKA-induced animal model of allergic asthma.

## 2. Materials and Methods

### 2.1. Animals

Balb/c female littermates (6 to 8 weeks of age, weighing 20–25 g) were purchased from Charles River Korea (OrientBio, Seungnam, Korea). All mice were kept under pathogen-free conditions using air conditioners and a 12-h light/dark cycle. In addition, all mice had free access to food and water during the experiments. The study was conducted according to the Rules for Animal Care and the Guiding Principles for Animal Experiments Using Animal by the University of Kyung Hee Animal Care and Use Committee. The protocols for the experimental procedures were approved by the Animal Welfare and Animal Care Committee of the University of Kyung Hee Animal Care and Use Committee.

### 2.2. Preparation of MMDT

**Figure 1:**
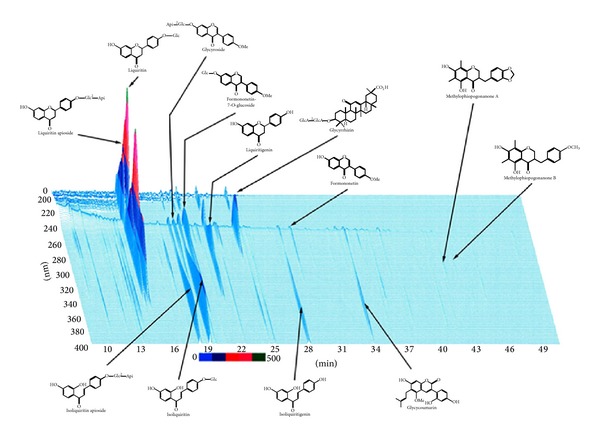
Three-dimensional HPLC profile of the methanol solution of Maekmoondong-tang. MMDT was analyzed by three-dimensional high-performance liquid chromatography (HPLC) and the ingredients were analyzed. MMDT was extracted with 30 mL of 50% methanol under ultrasonication for 30 min followed by centrifugation. The supernatant solution was analyzed by HPLC equipped with LC-10AD pumps, an SPD-M10Avp photodiode-array detector, and a CTO-10A column oven (Shimadzu, Kyoto, Japan) using a LS-120A column (250 4 : 6 mm, Tosoh, Tokyo, Japan). The representative HPLC profile was provided by Tsumura & Co., Tokyo, Japan.

Maekmoondong-tang (TJ-29, Lot: NGL 801) manufactured by Tsumura & Co. (Tsumura & Co., Tokyo, Japan) has been approved as a new drug by the Ministry of Food and Drug Safety (MFDS), Republic of Korea. MFDS accepted MMDT manufacturing process, efficacy and safety assurance, and quality control method and approved three-year validation period. This formula is a dried decoction of a mixture of six medicinal herbs (Table 1). MMDT (Table 1) were dissolved in the 12 times bigger weight/volume of water, boiled for 1 hour, filtered, and spray dried. The quality of each crude drug was tested in accordance with the pharmacopoeia of the Republic of Korea. Moreover, the test sample was analyzed by three-dimensional high-performance liquid chromatography (HPLC) and the ingredients were checked. MMDT was extracted with 30 mL of 50% methanol under ultrasonication for 30 min followed by centrifugation. The supernatant solution was analyzed by HPLC equipped with LC-10AD pumps, an SPD-M10Avp photodiode-array detector, and a CTO-10A column oven (Shimadzu, Kyoto, Japan) using a LS-120A column (250 4 : 6 mm, Tosoh, Tokyo, Japan). The solvents were (A) 0.05 M AcONH4-AcOH buffer (pH 3.6) and (B) 100% CH3CN. A linear gradient of 90% A and 10% B changing over 60 min to 0% A and 100% CH3CH was applied. A linear gradient of 90% A and 10% B changing over 60 min to 0% A and 100% B was applied (0% A and 100% B was continued for 20 min). The flow rate and the column temperature were 1.0 mL/min and 40°C, respectively. The UV data of the effluent from the column ranging from 200 to 500 nm were collected, and the peak analysis and assignment were performed using the system analysis software, CLASS-LC10 (Shimadzu) (Figure 1). For the administration of MMDT, the mice were treated with the MMDT extract (100, 200, 400 mg/kg/day) suspended in PBS by oral gavage before the intratracheal CKA challenge.

### 2.3. Experimental Protocol and Design

**Figure 2:**
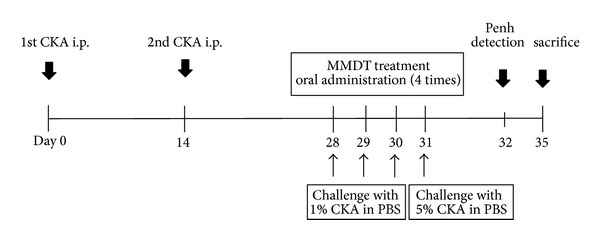
Schematic diagram of the experimental procedure. The mice were sensitized by intraperitoneal (i.p.) injection of CKA on day 0 and 14. Subsequently, the mice were intratracheally (i.t.) challenged with CKA for four consecutive days. On day 32, lung function was measured by whole body plethysmography, and tissue samples were collected on day 35.

The mice were randomly divided into six groups (*n* =  6~7 per group). The mice were sensitized by intraperitoneal (i.p.) injections with 10 *μ*g of CKA (Holister-Stier, Spokane, WA, USA) in incomplete Freund's adjuvant (Sigma-Aldrich, MO, USA) on day 0 and 14. Subsequently, the mice received an intratracheal (i.t.) challenge with CKA (1% CKA in phosphate-buffered saline (PBS)) on day 28 to 30 and 5% CKA in PBS on day 31 (Figure 2). The experiment schedule was modified from the methods of McGee and Agrawal [18]. The CKA-sensitized mice were treated with MMDT (100, 200, and 400 mg/kg/day) suspended in PBS by oral gavage 2 hr before the CKA challenge. Negative control (NC) and CKA-exposed (CKA) groups were treated with only PBS by oral gavage, and the positive control (MK) group was treated with Montelukast sodium (10 mg/kg/day) suspended in PBS by oral gavage before the CKA challenge. One day after last CKA challenge, the mice were analyzed using noninvasive lung function measurements (All Medicus, Seoul, Korea) to assess AHR. On day 35, mice were euthanized by intraperitoneal injection of pentobarbital sodium (50 mg/kg, Hanlim Pharm. Co., Seoul, Korea) and exsanguination without any previous intervention. The animal experiments were reported following guidelines recommended from the Animal Research: Reporting *In Vivo* Experiments (ARRIVE) (http://www.nc3rs.org.uk/page.asp?id=1357). We performed experiments two times independently to clarify that MMDT has significant effect on attenuation of cockroach allergen-induced airway responses.

### 2.4. Bronchoalveolar Lavage Fluid (BALF)

On day 35, all mice were sacrificed and BALF was collected. BALF was collected by infusion and extraction of 1 mL of ice-cold PBS. This was repeated three times, and the lavages were then pooled (mean volume, 2.0± mL). The recovered BALF (70–80%) was centrifuged at 13,000 rpm for 10 min. The cell pellets were resuspended in 1 mL of PBS, and the cells were adhered to glass slides using cytocentrifugation. The total viable cell counts were determined in a hemocytometer using trypan blue exclusion. The differential counts of eosinophils, neutrophils, lymphocytes, and macrophages were determined on cytospin smears of BALF samples (5 × 10^5^/200 *μ*L of cells) from individual mice stained with Diff-Quick staining (Life Technologies, Auckland, New Zealand) after counting 500 cells. The BALF was then centrifuged and the supernatants were kept at −70°C. The results are expressed as total cell number × 10^4^.

### 2.5. Assessment of Cytokines in BALF Using Enzyme-Linked Immunosorbent Assay (ELISA)

The concentration of Th2 cytokines (IL-4, IL-5, and IL-13) in the BALF were determined using a commercial enzyme-linked immunoassay kit (BD, San Diego, USA, for IL-4, IL-5, and R&D; Minneapolis, USA, for IL-13) according to the manufacturer's protocols. Detection lower limits for the IL-4, IL-5, and IL-13 ELISAs were 7.8, 15.62, and 62.5 pg/mL, respectively. All results were normalized to the total BALF protein amount in each sample [19].

### 2.6. Determination of IgE Titers Using ELISA

For serum, 96-well immunomicroplates (Costar, NY, USA) were coated with anti-mouse IgE monoclonal antibody. Serum was diluted (1 : 250) with 5% FBS in PBS (assay diluent), and IgE titers (BD Pharmingen) were measured using standardized sandwich ELISAs according to the manufacturer's protocol. The detection lower limit for the IgE ELISA was 1.5 ng/mL. The optical density was measured at 450 nm in a microplate reader (SOFT max PRO, version 3.1 software, CA, USA).

### 2.7. Histological Examination

Trachea and lung tissues were removed from the mice. The tissues were fixed in 4% paraformaldehyde, embedded in paraffin after dehydration, and then cut into 5 *μ*m sections. The sections were treated with hematoxylin and eosin (H&E), periodic acid-Schiff (PAS), and immunohistochemical stains, such as myosin light chain 2 (MLC2). The 5 *μ*m sections of the lower trachea and lung tissue were treated with 0.3% H_2_O-methanol for 20 minutes to block endogenous peroxidase. Subsequently, the sectioned tissues were incubated at 4°C overnight with an anti-MLC2 rabbit polyclonal antibody (1 : 50 dilution; Santa Cruz Biotechnology, CA, USA). After the slides were incubated with avidin-biotin peroxidase complex (ABC kit, Vestor Laboratories, CA, USA), the color was developed with 3,3′-diaminobenzidine tetrachloride (DAB; Zymed Laboratories, CA, USA). After immunohistochemical staining, the slides were counterstained with Harris's hematoxylin for 1 minute and then mounted with Canada balsam (Show Chemical Co. Ltd., Tokyo, Japan).

### 2.8. Measuring Airway Hyperresponsiveness (AHR) to Methacholine

Noninvasive measurement of airway responsiveness was used in this study (All Medicus, Seoul, South Korea). Airway responsiveness was assessed in unrestrained and conscious mice at 24 hours after last challenge with CKA, as described in other studies [20–22]. Each mouse was placed in a plastic chamber and exposed to increasing concentrations of aerosolized methacholine (Sigma-Aldrich, MO, USA). The mice were placed in a barometric plethysmographic chamber and baseline readings were taken for 3 minutes. The enhanced pause (Penh) was calculated according to the manufacturer's protocol (i.e., (expiratory time/relaxation time − 1) × (peak expiatory flow/peak inspiratory flow)). Penh is a dimensionless parameter that represents a function of the proportion of maximal expiratory to maximal inspiratory box pressure signals and a function of the timing of expiration. The results are expressed as the percent increase in Penh following the challenge with each concentration of methacholine (0, 25, 50, and 100 mg/mL).

### 2.9. Statistical Analysis

The statistical analysis of the data was conducted using the Prism 5 software (Graph Pad Software Inc., CA, USA). All of the values are presented as the mean ± S.E.M (standard error of the mean). Statistical differences in the mean values among treatment groups were determined using a one-way ANOVA followed by Newman-Keuls Multiple comparison test. In all cases, *P* < 0.05 was considered to be statistically significant.

## 3. Results

### 3.1. The Effects of MMDT on Pulmonary Inflammation in CKA-Induced Allergic Asthmatic Mice

**Figure 3:**
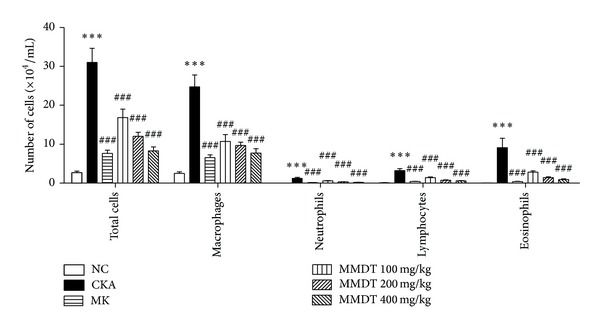
The effect of MMDT extract on immune cell profiles in BALF. The effects of MMDT extracts on the recruitment of inflammatory cells into BALF in CKA-induced allergic asthmatic mice. The MMDT- and MK-treated groups received 100, 200, and 400 mg/kg MMDT and 10 mg/kg MK orally between days 28 and 31. Control and CKA-immunized mice were treated with saline on the same days. The BALF cells were separated using a hemacytometer and then stained with Diff-Quick; 500 cells were counted. Total leukocytes from the BALF were counted. Normal control mice treated with PBS only (NC), CKA-challenged mice treated with PBS (CKA), CKA-challenged mice treated with 10 mg/kg of Montelukast (MK), CKA-challenged mice treated with 100 mg/kg of MMDT (MMDT 100 mg/kg), CKA-challenged mice treated with 200 mg/kg of MMDT (MMDT 200 mg/kg), and CKA-challenged mice treated with 400 mg/kg of MMDT (MMDT 400 mg/kg). The data are shown as the mean ± S.E.M. Statistical analysis was conducted by one-way ANOVA followed by the Newman-Keuls Multiple Comparison test (significantly different from NC, **P < 0.01, ***P < 0.001; significantly different from CKA, ^##^ *P* < 0.01, ^###^ *P* < 0.001, *n* = 6-7).

To evaluate the effects of MMDT on pulmonary inflammation, the immune cells were analyzed in pneumonocytes of CKA-induced allergic asthmatic mice. A significant increase in the total number of cells was observed in the CKA group compared with the Montelukast (MK-)treated (10 mg/kg) and MMDT-treated (100, 200, or 400 mg/kg) groups. In addition, the influx of macrophages, eosinophils, neutrophils, and lymphocytes was remarkably higher in the CKA group compared with the MK-treated (10 mg/kg body wt) and MMDT-treated (100, 200, or 300 mg/kg body wt) groups (Figure 3). Our study shows twice as many macrophages as eosinophils in the BALF that lies on the line of other researchers' results [23–25].

### 3.2. The Effect of MMDT on IgE Production and Th2 Inflammatory Cytokines Secretion

**Figure 4:**
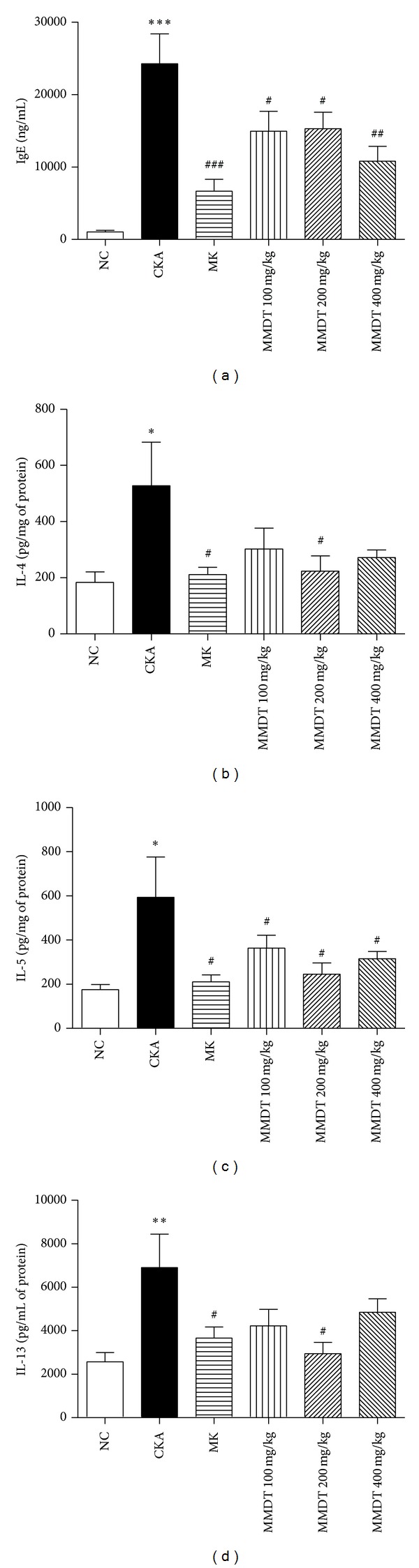
Total IgE production from serum and Th2 cytokine secretion from BALF in CKA-induced asthmatic mice. Blood was collected from the retroorbital plexus. The BALF was collected by infusion and extraction by ice-cold PBS. Total IgE and Th2 cytokine (IL-4, IL-5, and IL-13) levels were measured by ELISA. Normal control mice treated with PBS only (NC), CKA-challenged mice treated with PBS (CKA), CKA-challenged mice treated with 10 mg/kg of Montelukast (MK), CKA-challenged mice treated with 100 mg/kg of MMDT (MMDT 100 mg/kg), CKA-challenged mice treated with 200 mg/kg of MMDT (MMDT 200 mg/kg), and CKA-challenged mice treated with 400 mg/kg of MMDT (MMDT 400 mg/kg). The data are shown as the mean ± S.E.M. Statistical analysis was conducted by one-way ANOVA followed by the Newman-Keuls Multiple Comparison test (significantly different from NC, *P < 0.05, **P < 0.01, ***P < 0.001; significantly different from CKA, ^#^ *P* < 0.05, ^##^ *P* < 0.01, ^###^ *P* < 0.001, *n* = 6-7).

We also evaluated serum IgE production and Th2 inflammatory molecules generated by CKA-induced allergic asthma to ensure that MMDT inhibited the secretion of IgE and BALF inflammatory cytokines IL-4, IL-5, and IL-13 (Figure 3). In 100 and 400 mg/kg of MMDT, the level of IL-4 or IL-13 in BALF has tendency to reduce. However, 200 mg/kg of MMDT has significant effect on decrease of the level of IL-4 or IL-13 in BALF, which means that MMDT inhibit the secretion of IL-4 or IL-13. The total serum IgE titers and the secretion of cytokines IL-4 and IL-5 were significantly elevated in all of the CKA-sensitized and challenged mice compared with the control mice. As expected, the MMDT-treated mice exhibited reduced levels of serums IgE, IL-4, IL-5, and IL-13 compared with the CKA group (Figure 4).

### 3.3. The Effect of MMDT on Histological Changes in Lung Tissue

**Figure 5:**
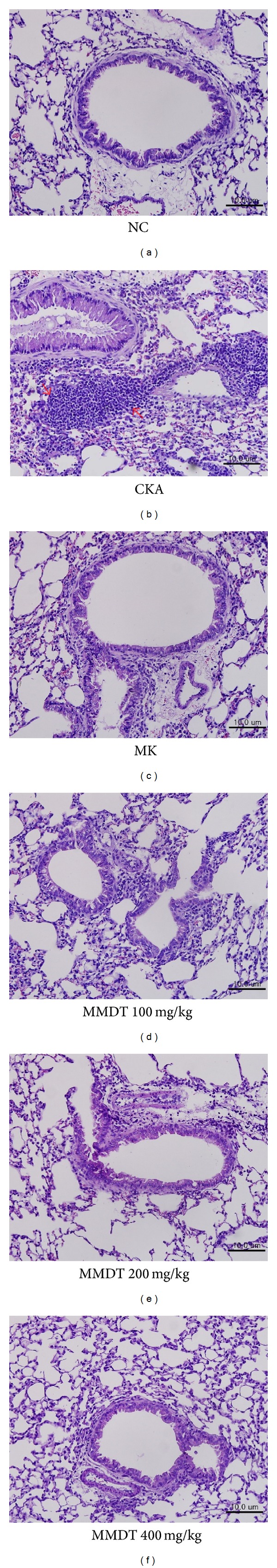
The effect of MMDT on eosinophils in airway inflammation. Balb/c mice were sensitized and challenged with CKA. Lung sections were stained with H&E and examined using light microscopy (magnification 200x). Normal control mice treated with PBS only (NC), CKA-challenged mice treated with PBS (CKA), CKA-challenged mice treated with 10 mg/kg of Montelukast (MK), CKA-challenged mice treated with 100 mg/kg of MMDT (MMDT 100 mg/kg), CKA-challenged mice treated with 200 mg/kg of MMDT (MMDT 200 mg/kg), and CKA-challenged mice treated with 400 mg/kg of MMDT (MMDT 400 mg/kg).

**Figure 6:**
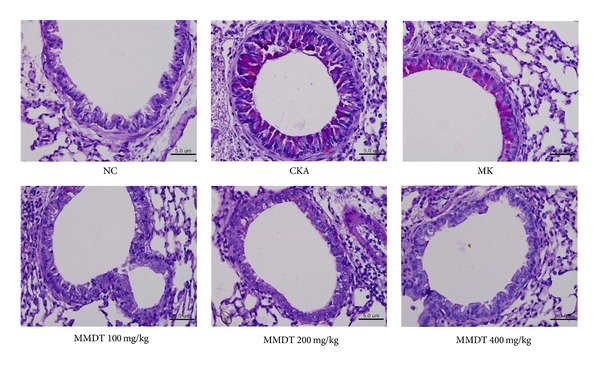
The effect of MMDT extract on goblet cells in lung tissues. The mucus substances were stained magenta by the PAS reaction (magnification 400x).

**Figure 7:**
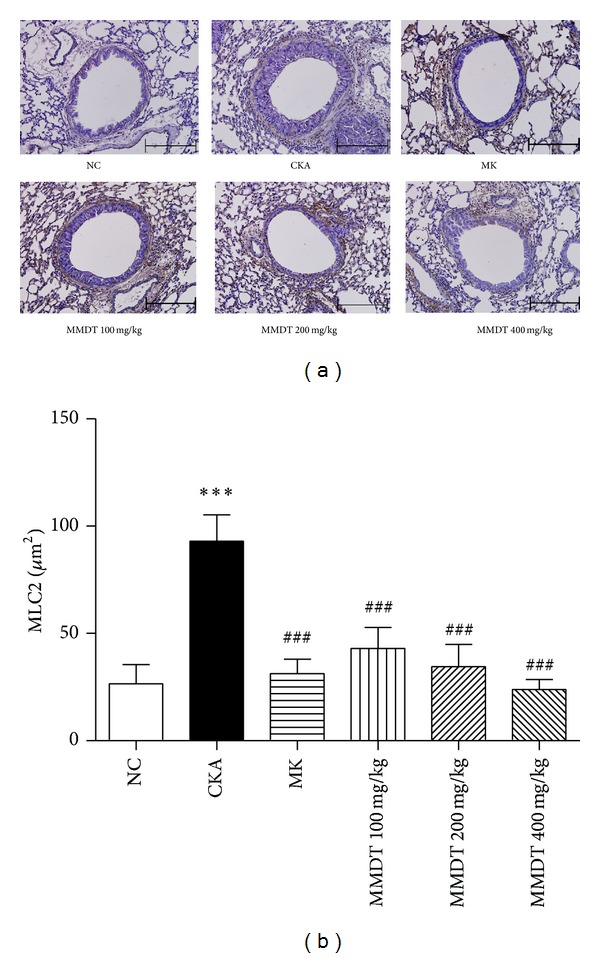
Effects of MMDT treatment on airway remodeling in lung tissue (MLC2 immunohistochemistry). (a) Stainedmouse lung sections and (b) MLC2 area (*μ*m^2^). Mouse lung sections were stained for MLC2 detection. The sections were incubated at 4°C overnight with an anti-MLC2 goat polyclonal antibody (1 : 50). The slides were then incubated with avidin-biotin peroxidase complex, and color was developed using 3,3-diaminobenzidine tetrachloride. The arrows indicate MCL2 positive cells (magnification 200x). Normal control mice treated with PBS only (NC), CKA-challenged mice treated with PBS (CKA), CKA-challenged mice treated with 10 mg/kg of Montelukast (MK), CKA-challenged mice treated with 100 mg/kg of MMDT (MMDT 100 mg/kg), CKA-challenged mice treated with 200 mg/kg of MMDT (MMDT 200 mg/kg), and CKA-challenged mice treated with 400 mg/kg of MMDT (MMDT 400 mg/kg). The data are shown as the mean ± S.E.M. Statistical analysis was conducted by one-way ANOVA followed by the Newman-Keuls Multiple Comparison test (significantly different from NC, **P < 0.01, ***P < 0.001; significantly different from CKA, ^##^ *P* < 0.01, ^###^ *P* < 0.001, *n* = 6-7).

H&E, PAS, and MLC2 immunohistochemical staining were performed on the lung tissues to analyze the effects of MMDT on histological features of asthma. The histological sections of lung tissue from CKA-exposed mice exhibited airway inflammation and were found to have infiltrating eosinophils in the peribronchial regions of the lung. Conversely, airway inflammation was inhibited in the histological sections of lung tissue obtained from MMDT-treated mice (Figure 5). PAS-positive mucus-counting goblet cells around the bronchial airway were detected in the CKA-treated group. However, the MMDT treatment substantially reduced the PAS-positive goblet cells around the bronchial airway (Figure 6). The CKA-challenged mice demonstrated expression of MLC2 in the peribronchial muscle layer of the lung, and the MMDT treatment abrogated the expression of this protein. The MLC2 positive areas of each group were calculated as well. All of the MLC2 positive areas were decreased significantly compared with the CKA group (Figure 7).

### 3.4. Inhibitory Effect of MMDT on AHR

**Figure 8:**
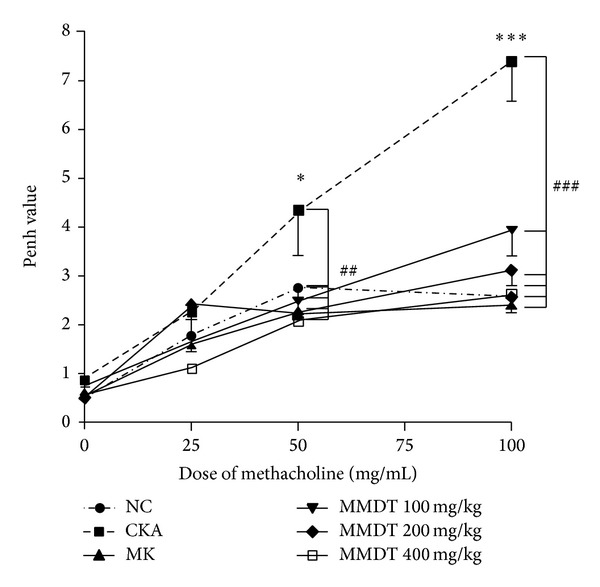
The effect of MMDT treatment on AHR to aerosolized methacholine. Penh was measured with OCP-3000. The mice were administered MMDT (100, 200, and 400 mg/kg) and MK (10 mg/kg) as described in materials and methods. The airway responsiveness to aerosolized methacholine was measured 24 h after the last CKA challenge. Normal control mice treated with PBS only (NC), CKA-challenged mice treated with PBS (CKA), CKA-challenged mice treated with 10 mg/kg of Montelukast (MK), CKA-challenged mice treated with 100 mg/kg of MMDT (MMDT 100 mg/kg), CKA-challenged mice treated with 200 mg/kg of MMDT (MMDT 200 mg/kg), and CKA-challenged mice treated with 400 mg/kg of MMDT (MMDT 400 mg/kg). The data are shown as the mean ± S.E.M. Statistical analysis was conducted by one-way ANOVA followed by the Newman-Keuls Multiple Comparison test (significantly different from NC, *P < 0.05, **P < 0.01, ***P < 0.001; significantly different from CKA, ^#^ *P* < 0.05, ^##^ *P* < 0.01, ^###^ *P* < 0.001, *n* = 6-7).

To evaluate the inhibitory effect of MMDT on airway hyperresponsiveness, whole body barometric plethysmographic analysis was performed. The Penh values increased significantly in the CKA group compared with the control group at 100 mg/mL methacholine (7.4 ± 2.1 versus 2.5 ± 0.6, resp.). Also, the Penh values significantly inhibited in the 100 mg/kg, 200 mg/kg, and 400 mg/kg MMDT groups with the administration of 50 mg/mL (4.3 ± 0.9 versus 2.4 ± 0.2, 4.3 ± 0.9 versus 2.2 ± 0.1, and 4.3 ± 0.9 versus 2.0 ± 0.1, resp.) and 100 mg/mL (7.4 ± 2.1 versus 3.9 ± 1.4, 7.4 ± 2.1 versus 3.1 ± 0.7, and 7.4 ± 2.1 versus 2.6 ± 0.9, resp.) of methacholine compared with the CKA group (Figure 8).

## 4. Discussion

Asthma is a complex disease that is characterized by reversible airway obstruction, elevated serum levels of IgE, airway eosinophilia, airway remodeling, mucus hypersecretion, and AHR to bronchospasmogenic stimuli [5, 6, 26]. Current standard medications involve combination therapies, which include inhaled corticosteroids, leukotriene receptor antagonists (e.g., Montelukast, MK), *β*2-agonists, and others [2, 27]. However, these therapies can produce potential negative side effects and do not consistently ameliorate airway inflammation in many asthmatic individuals [3, 7, 8]. Therefore, there is a need for the development of safe and efficacious treatments [9]. Medicinal plants have long been used as an excellent source of anti-inflammatory agents with minimal side effects and are successfully employed in inflammation treatment as harmless alternatives to conventional medicine. Thus, the raw materials of such products have been used to develop new drugs recently [28–30].

MMDT has been used traditionally to treat chronic respiratory diseases, such as asthma. In our cockroach extract-induced allergic asthma models, the effects of MMDT on lung inflammation were similar to the effects of MK. There have been many studies conducted to identify the potential efficacy of MMDT in asthma [13, 14, 31–33]. Park and Seo [33] and Ryu et al. [31] showed that Kami-MMDT and MMDT reduce malformed respiration and eosinophil infiltration. Kim and Han [13] showed that the contractile force of acetylcholine from tracheal smooth muscle in control rats was significantly inhibited by MMDT. Similarly, Tamaoki et al. [14] demonstrated that the beta-adrenergic function of MMDT lessened the tension of bronchial smooth muscle. Immunological experiments have been conducted as well. Kim et al. [11] showed that MMDT increased T cells, especially Th1 cells and IFN-r, but had no effect on IL-4. Moreover, Kim et al. [12] observed that MMDT regulated total cell, lymphocyte, CD4^+^ T cells, CD8^+^ T cells in BALF, and serum IgE levels. Additionally, Aizawa et al. [15] concluded that MMDT significantly inhibited airway hyperresponsiveness but had no effect on neutrophil influx or plasma extravasation. In clinical studies, Hsu et al. [16] and Watanabe et al. [17] demonstrated that MMDT could improve the symptoms of asthma.

However, with these studies, the underlying mechanisms of how MMDT functions to ameliorate allergic asthma were evaluated in detail. Airway inflammation is a characteristic feature of human asthma. In humans with asthma, morphological and inflammatory changes are accompanied by increases in lung IgE, IL-4, IL-5, and IL-13. Specifically, IgE production is a hallmark of allergic diseases [34, 35]. IL-4, IL-5, IL-6, and IL-13 can enhance IgE production. In addition, IL-13 is both necessary and sufficient to induce AHR [36–40]. AHR is closely associated with eosinophilia, as evidenced by an increase in the number of eosinophils in BALF [10, 41]. In the present study, MMDT treatment significantly reduced the total cells and the influx of macrophages, eosinophils, neutrophils, and lymphocytes in the CKA-treated control group. Serum IgE production and Th2 cytokines, such as IL-4, IL-5, and IL-13, were also reduced, although these were not shown at all concentrations (100, 200, and 400 mg/kg). The results of this study showed that treatment with 200 mg/kg of MMDT shows inhibitory effects on CKA-induced allergic asthma in mice better than 100 mg/kg of MMDT as shown with several biomarkers. For example, 200 mg/kg of MMDT showed significant decrease of the level of IL-4 and IL-13 in BALF, but treatment with 100 or 400 mg/kg of MMDT groups did not. These results suggest that treatment with 200 mg/kg of MMDT is generally more effective than 100 or 400 mg/kg of MMDT in CKA-induced allergic asthma. IL-13 is a pleiotropic cytokine produced mainly by T cells and is critical for the development of airway hyperresponsiveness (AHR) associated with allergen exposure [42]. However, IL-13 was not the only cytokine associated with AHR. For example, Schwarze et al. showed that the lack of IL-5 is the critical element for eosinophil recruitment and for the development of AHR in response to RSV infection and, in turn, the absence of IL-5 appeared protective against the development of AHR [43]. For these reasons, AHR data in this study showed suitable results; nevertheless, the level of IL-13 was only slightly suppressed. In addition, MMDT produced histological changes in the lung tissue. MMDT inhibited infiltrating eosinophils in the peribronchial regions and PAS-positive goblet cells around the bronchial airway. Additionally, the expression of MLC2 in the peribronchial muscle layer of the lung was abrogated. These findings demonstrate that MMDT has the potential to counteract allergic asthma-associated airway remodeling. Furthermore, the Penh values were significantly reduced after applying MMDT. These data demonstrated that MMDT had an inhibitory effect on AHR.

Several herbal medicines, including Ophiopogon japonicas, Pinellia ternata, and Panax ginseng, have been used for the treatment of asthma [44–46]. In study of Lee et al., Pinellia ternata treatment did not reduce influx of macrophages and neutrophils on OVA-induced mice model [46]. However, our study showed that MMDT treatment significantly reduced the influx of macrophages and neutrophils in the CKA-treated control group. Herbal formulas were widely used as traditional medicines to treat many different types of disease [47, 48]. MMDT was one of these traditional herbal medicines and has been widely used to treat respiratory diseases. In experimental studies, MMDT reduced malformed respiration and eosinophil infiltration and released the tension of bronchial smooth muscle [12, 14]. Additionally, MMDT had immunomodulatory action and regulated many immune cytokines to reduce hypersensitivity. The components of MMDT have been shown to have various biological effects. *Ophiopogon japonicus*, a major component of MMDT, possesses antimyocardial ischemia effects, antianaphylaxis effects, and anti-inflammatory effects, decreases blood sugar, and boosts immune activities, which are effective in treating thrombosis, myocardial ischemia, arrhythmias, respiratory disease, and hyperglycemia [44, 49, 50]. Hung et al. showed that the release of the inflammatory chemokine eotaxin, stimulated by IL-4 and the combination of IL-4 and TNF-*α* in BEAS-2B cells, mimics the *in vivo* conditions in bronchial allergic asthma. In addition, this herb has been reported to suppress the production of NO in the murine microglial cell line BV-2 [44]. *Pinellia ternata* has been applied for antiemetic, antitussive, sedative, and anti-inflammatory purposes [45]. *Pinellia ternata*, another component of MMDT, potentially inhibits TNF-*α*-induced NF-*κ*B activation and possesses agonistic activity toward the peroxisome proliferator-activated receptors (PPAR) PPAR*α* and PPAR*γ* [51]. The NF-*κ*B pathway and PPARs are important drug targets with regard to inflammation and metabolic dysfunction. The NF-*κ*B pathway is a key regulator of inflammation [52, 53]. PPAR*α* and PPAR*γ* agonism is also linked to the suppression of proinflammatory genes via interference of the NF-*κ*B pathway [54, 55]. Lee et al. showed that *Pinellia ternata* markedly suppressed the OVA-induced iNOS expression in the lungs, which may be the result of inhibition of downstream NF-*κ*B activity and the reduced level of IL-13 in the allergic airways [46]. *Oryza sativa* reduces the risk of hepatic fat accumulation and improves insulin resistance in high-fat diet-fed mice [56]. *Zizyphus jujuba *possesses health-promoting effects, such as protecting the gastrointestinal tissue from protein inflammatory injury and antiproliferative and apoptotic effects in human breast cancer cells [57, 58]. *Panax ginseng*, one of the most well-known medicinal herbs, has been used in traditional Korean medicine as a herbal remedy for various disorders [59]. *Panax ginseng *possesses immunomodulatory, antitumor, antioxidant, antiradiation, antiadhesive, and hypoglycemic activities [60–63]. Kim and Yang showed that inhibition of MAP kinases regulated via CD40 ligation by *Panax ginseng* treatment may inhibit the production of inflammatory cytokines and serves as a mechanism of its antiallergic and antiasthmatic activity [59]. *Glycyrrhiza uralensis *has been used for detoxification, moistening the lungs, relieving coughs, reducing inflammation, and the treatment of gastric ulcers [64]. Lee et al. showed that *Glycyrrhiza uralensis* inhibit the inflammatory process in lung tissue through suppression of the NF-*κ*B signaling pathway [65]. The biological activities of compounds isolated from *Glycyrrhiza uralensis *have antitussive, antiallergic, anticancer, and anti-inflammatory activities [66–68]. The therapeutic potency of MMDT should be attributed to its combined and synergistic effects on multiple targets as a result of its diverse components.

In summary, during the allergic sensitization procedure, MMDT inhibited AHR through the reduction of allergy-related inflammation and caused histologic changes in airway tissues.

However, IL-4 and IL-13 levels were not reduced significantly at each concentration of MMDT; Kim et al. also reported that IL-4 levels were not affected by MMDT [11]. However, our data showed that 200 mg/kg of MMDT regulated IL-4 levels significantly. Therefore, the addition of mice to the study may reveal more clearly the potential of MMDT to reduce IL-4 levels. MMDT was as effective as the positive control (MK) in most experiments. This study provides reasonable evidence that MMDT could be developed as an alternative medication for clinical use.

## 5. Conclusions

Taken together, the results of this study suggest that MMDT significantly reduces CKA-induced lung inflammation by suppressing peribronchial and perivascular inflammatory cell infiltrates. Additionally, MMDT significantly inhibits increased AHR. Both the anti-inflammatory effects and the effects on AHR indicate that MMDT possesses a therapeutic potential for the treatment of asthma. Therefore, further studies should be conducted to aid in the discovery of new therapeutic agents for the treatment of allergic asthma.

## Figures and Tables

**Table 1:** The contents of MMDT.

Herbal medicines	Ratio
Ophiopogon japonicus	10
Pinellia ternata	5
Oryza sativa	5
Zizyphus jujuba	3
Panax ginseng	2
Glycirrhiza uralensis	2
